# Robust Modeling of Differential Gene Expression Data Using Normal/Independent Distributions: A Bayesian Approach

**DOI:** 10.1371/journal.pone.0123791

**Published:** 2015-04-24

**Authors:** Mojtaba Ganjali, Taban Baghfalaki, Damon Berridge

**Affiliations:** 1 School of Biological Science, Institute for Research in Fundamental Sciences (IPM), Tehran, Iran; 2 Department of Statistics, Faculty of Mathematical Sciences, Shahid Beheshti University, Tehran, Iran; 3 Department of Statistics, Faculty of Mathematical Sciences, Tarbiat Modares University, Tehran, Iran; 4 Farr Institute-CIPHER, College of Medicine, Swansea University, Swansea, Wales, U.K.; Irvine, UNITED STATES

## Abstract

In this paper, the problem of identifying differentially expressed genes under different conditions using gene expression microarray data, in the presence of outliers, is discussed. For this purpose, the robust modeling of gene expression data using some powerful distributions known as normal/independent distributions is considered. These distributions include the Student’s t and normal distributions which have been used previously, but also include extensions such as the slash, the contaminated normal and the Laplace distributions. The purpose of this paper is to identify differentially expressed genes by considering these distributional assumptions instead of the normal distribution. A Bayesian approach using the Markov Chain Monte Carlo method is adopted for parameter estimation. Two publicly available gene expression data sets are analyzed using the proposed approach. The use of the robust models for detecting differentially expressed genes is investigated. This investigation shows that the choice of model for differentiating gene expression data is very important. This is due to the small number of replicates for each gene and the existence of outlying data. Comparison of the performance of these models is made using different statistical criteria and the ROC curve. The method is illustrated using some simulation studies. We demonstrate the flexibility of these robust models in identifying differentially expressed genes.

## 1 Introduction

Microarrays allow the simultaneous measurement of the expression levels of thousands of genes. This excellent data structure has inspired a completely new area of research in statistics and bioinformatics [1]. [2] considered the problem of identifying differentially expressed genes under different conditions using gene expression microarray data. They used a robust Bayesian hierarchical model for testing hypotheses relating to different gene expressions. Before this, an initial statistical treatment was given by [3] to detect differentially expressed genes. Variants of t or F-statistics were used by [4]. A modification of the t-statistic was used by [5, 6]. [7] using a permutation technique, estimated and controlled false discovery rate (FDR). An empirical Bayes approach was used by [8–10]. More fully Bayesian approaches, using Markov Chain Monte Carlo (MCMC), were applied by [11, 12]. [2, 13] introduced a hierarchical t distribution formulation which is more robust to outliers than the normal model. [2] call their model BRIDGE (Bayesian Robust Inference for Differential Gene Expression). BRIDGE (2013, http://www.rglab.org) has been recently constructed as a package consisting of several functions in R software for testing differential expressions in multiple samples.

[14] introduced a Laplace mixture model as a long-tailed alternative to the normal distribution when identifying differentially expressed genes in microarray experiments. This model permits greater flexibility than models in current use as it has the potential, at least with sufficient data, to accommodate both whole genome and restricted coverage arrays. The Laplace model appears to give some improvement in fit to data. [15] also emphasized the potential insufficiency of the Gaussian noise model in microarray data analysis and proposed different noise models. In their work the goodness of fit of noise models is quantified by a hierarchical Bayesian analysis of variance model, which predicts normalized expression values as a mixture of a Gaussian density and t-distributions with adjustable degrees of freedom. They find that, irrespective of the chosen preprocessing and normalization method, a heavy-tailed noise model is a better fit than a simple Gaussian. [16] discussed robust nonlinear differential models of gene expression. Also, variance-modeling considerations for robust data analysis were emphasized by [17].

In the current paper, an extension of the Bayesian hierarchical model of [2, 13, 18] is proposed using the family of normal/independent (N/I) distributions for errors to achieve some more robust models for analyzing gene expression microarray data. Our approach will let the data themselves determine the best robust model. This family includes normal and t distributions as well as slash, Laplace and contaminated normal distributions.

The same as [2] the model includes an exchangeable prior for the variances, allowing each gene to have a different variance and a prior for the model that allows us to detect differentially expressed genes in multiple-sample experiments. In practice, the prior is a mixture of singular Gaussian distributions. Inference is based on the posterior probabilities of differential expressions calculated from the chosen model. We call our method BRIN/IDGE (Bayesian Robust Inference using N/I family for Differential Gene Expression). Parameter estimation is carried out using Markov Chain Monte Carlo. The method is illustrated using two publicly available gene expression data sets which are fully explored in the next section. Also, some simulation studies are conducted in order to illustrate the proposed approach.

[2] compared their BRIDGE method for testing differentially expressed genes with other methods: (i) the t-test, (ii) the Bonferroni-adjusted t-test, (iii) significance analysis of microarrays (SAM, [7]), (iv) empirical Bayes lognormal-normal and (v) gamma-gamma models [8] and (vi) Efron’s empirical model [10]. In this paper, not only we will compare the performance of members of BRIN/IDGE with these six methods, but also we will compare the performance of different members of BRIN/IDGE, and will find which one provides the best fit to two-sample and multiple-sample data sets.

This article is organized as follows. Section 2 introduces the data sets and some notation. In Section 3 we give an overall view of normal/independent (N/I) distributions. In Section 4, we present the Bayesian hierarchical model using the N/I structure. In Section 5, we apply the proposed models to the two datasets introduced in Section 2 and test the differential expressions using different members of the family of N/I distributions and compare the performance of these models based on Bayesian false discovery rate (bFDR), Bayesian true negative rate (bTNR), Bayesian false negative rate (bFNR) and area under the curve (AUC). Section 6 contains the results of some simulation studies. In the final section we present some conclusions. Also, more details of members of the normal/independent (N/I) distributions and an analysis of Bayesian false discovery rate are given in appendices A and B, respectively.

## 2 Data

### 2.1 Golub data

Gene expression data (3051 genes and 38 tumor mRNA samples) are extracted from the leukemia microarray study of [19]. Pre-processing was done as described in [4]. The challenge of cancer treatment has been to target specific therapies to pathogenetically distinct tumor types in order to maximize efficacy and minimize toxicity. [19] chose acute leukemias as a test case. They classified acute leukemias as those arising from lymphoid precursors (acute lymphoblastic leukemia, ALL) or from myeloid precursors (acute myeloid leukemia, AML). The leukemia data set consisted of 38 bone marrow samples (27 ALL, 11 AML) obtained from acute leukemia patients at the time of diagnosis. RNA prepared from bone marrow mononuclear cells was hybridized to high-density oligonucleotide microarrays, produced by Affymetrix and contained probes for 3051 human genes. For each gene, a quantitative expression level is available. The data take the form *Y*_*isr*_, *i* = 1, 2, …, *N*; *s* = 1, 2 and *r* = 1, 2, …, *n*_*s*_, where *Y*_*isr*_ is the log transformed estimated intensity for gene *i* in group *s* from replicate *r*.

**Fig 1:**
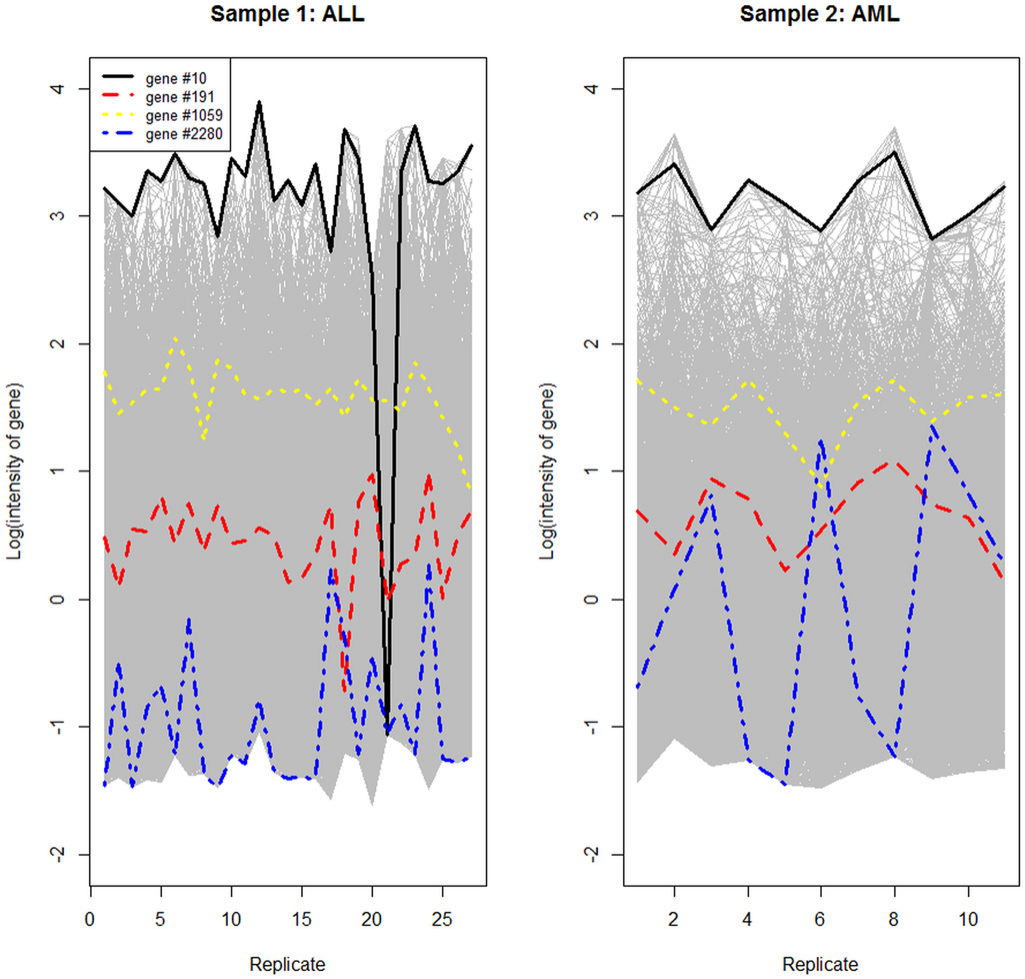
Profiles of log transformed estimated intensities from each group. Left panel for ALL group and right panel for AML group.

Fig 1 displays profiles of the log transformed estimated intensities against replicate number for the two subsets of Golub data. Also, the profiles of four randomly selected genes are also drawn (genes 10, 91, 1059 and 2280). These profiles show that, for example, gene #2280 may be identified as being expressed differentially between the two groups (ALL and AML). The profiles of genes and #191 and #1059 in the two groups have similar behavior; thus, these genes may not be identified as differentially expressed genes. Also, this figure shows that the log transformed estimated intensities for some genes (for example, gene #10) include outliers (for example, replicate 21 in the ALL group).

### 2.2 The hereditary breast cancer data

Many cases of hereditary breast cancer are due to mutations in either the BRCA1 gene or the BRCA2 gene. The histopathological changes in these cancers are often characteristic of the mutant gene. We hypothesize that the genes expressed by these two types of tumor are also distinctive, perhaps allowing us to identify cases of hereditary breast cancer on the basis of gene-expression profiles. [20] conducted a study to examine breast cancer tissues from patients carrying mutations in the predisposing genes, BRCA1 or BRCA2, or from patients not expected to carry a hereditary mutation.

[20] examined RNA from samples of primary tumors from seven carriers of the BRCA1 mutation, eight carriers of the BRCA2 mutation, and seven patients with sporadic cases of breast cancer. In these data, samples or groups refer to tissue sample types and there is no color swap. A set of 3226 genes was pre-selected by [20] by filtering the raw images. The data take the form *Y*_*isr*_ ≡ *log*_2_(*x*_*isr*_/*ref*_*ir*_), *i* = 1, …, *N*; *r* = 1, …, *n*_*s*_; *s* = 1, 2, 3, where *x*_*isr*_ is the intensity from gene *i* of the *r* ^*th*^ (biological) replicate in group *s*, and *ref*_*ir*_ is the intensity from a common reference sample. Note that here, [20] used a reference sample because there are three groups of interest: BRCA1 mutation, BRCA2 mutation, and sporadic cases of breast cancer.

**Fig 2:**
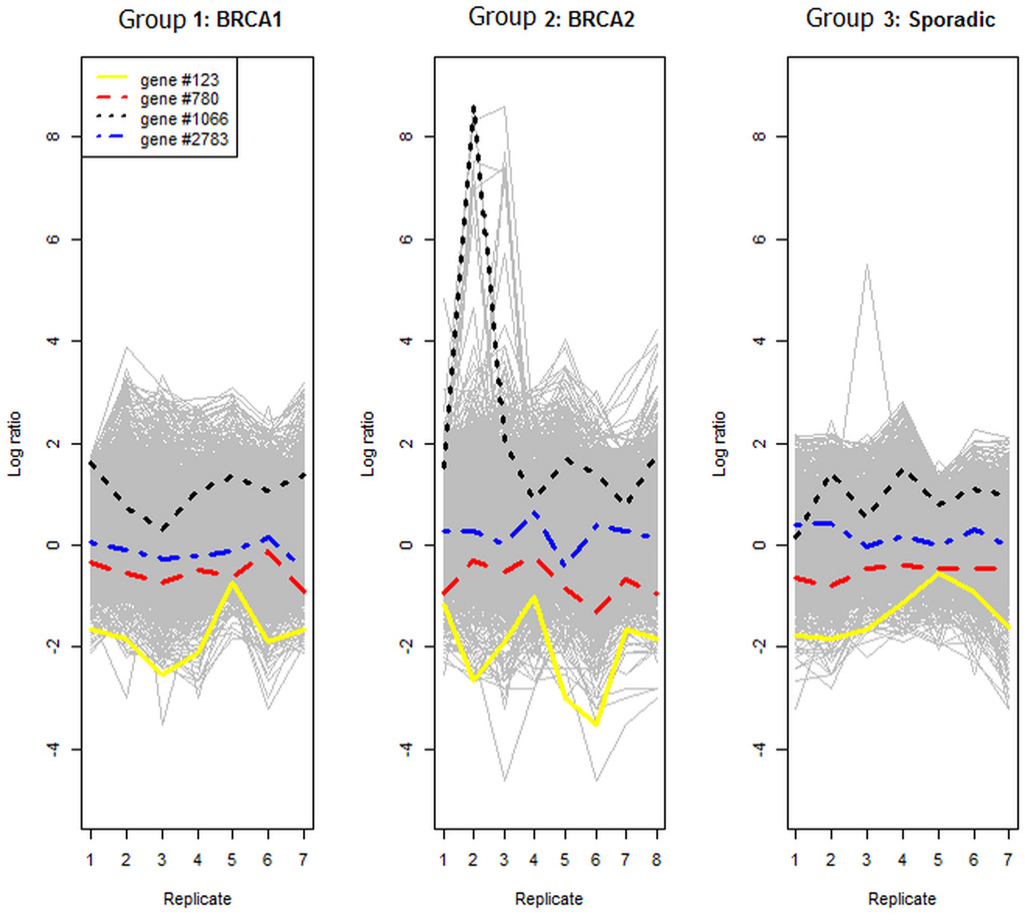
Profiles of log ratios from each group. Left panel for BRCA1, middle panel for BRCA2 and right panel for sporadic case.

Fig 2 displays the gene-expression profiles (log ratios) against the replicate number of tumors with BRCA1 mutations, tumors with BRCA2 mutations, and sporadic tumors. This figure shows that there are some differences, particularly in terms of the variation in log ratios between breast tumors with BRCA2 mutations and those with other mutations. There is greater variation in log ratios for the BRCA2 than for the other two mutations. Four randomly selected genes are highlighted in different linestyles. They allow us to follow the behaviour of the randomly selected genes in the three groups. Also, this figure shows that some genes (for example, gene #1066) include outliers in their replications (for example, replicate 2 in group 2).

## 3 Normal/independent (N/I) distribution

A normal/independent (N/I) distribution [21] is a stochastic representation of the random variable $Y=\mu +e/\sqrt{u}$, where *μ* is a location parameter, *u* is a positive random variable, with density *g*(*u*; ***ν***), where ***ν*** is a scalar or random vector of parameters, and error (*e*) is a normally distributed random variable with mean 0 and variance *σ*.

Given *u*, *Y* follows a normal distribution with location parameter *μ* and scale parameter *u* ^−1^ *σ*. Then, the marginal distribution of *Y* is $f(y|\mu ,\sigma ,\nu )=\int_{0}^{\infty }\phi (y;\mu ,u^{-1}\sigma )dG(u;\nu ),$ where *ϕ*(.; *μ*, *σ*) is the density function of *N*(*μ*, *σ*) and the *G*(*u*; ***ν***) is the distribution function of *u*.

The class of N/I distributions includes the *t*, the slash, the contaminated normal, and the Laplace distributions. All these distributions have heavier tails those of the normal distribution, and can be used for robust inference. These distributions are described in S1 Appendix.

## 4 Bayesian Robust Inference using N/I family for Differential Gene Expression (BRIN/IDGE)

We consider two scenarios for modeling differential gene expression under N/I distributional assumptions. One is for a two-sample case and the other introduces for a multiple-sample case. Let *Y*_*isr*_, *i* = 1, 2, …, *N*; *r* = 1, 2, …, *n*_*s*_ and *s* = 1, 2, …, *k* be the gene expression data for gene *i* from replicate *r* in sample *s*.

### 4.1 Two-group case

The simplest method for model comparison of two samples is indirect comparison and oligonucleotide arrays [2]. In this scenario, the model can be modified as follows: $Y_{isr}=\mu_{is}+{ɛ}_{isr}/\sqrt{u_{isr}},$ where ${ɛ}_{isr}|\tau_{ɛi}∼N(0,\tau_{ɛi}^{-1})$ and *U*_*isr*_ ∼ *g*(*u*_*isr*_; ***v**_i_*).

In this scenario, ***μ***_*i*_ = (*μ*_*i*1_, *μ*_*i*2_) is modeled with a mixture of two normal distributions as follows:

$$\begin{array}{ccc} \mu_{i}|\tau_{\mu },p & ∼ & \left(1-p\right)N\left(\mu_{i1};0,\tau_{\mu 12}^{-1}\right)I_{\left[\mu_{i1}=\mu_{i2}\right]}\\ & & +\;pN\left(\mu_{i1};0,\tau_{\mu 1}^{-1}\right)N\left(\mu_{i2};0,\tau_{\mu 2}^{-1}\right)I_{\left[\mu_{i1}\neq \mu_{i2}\right]}, \end{array}$$ (1)

where ***τ***_*μ*_ = (*τ*_*μ*1_, *τ*_*μ*_2__, *τ*_*μ*12_). Also, $N(\mu_{i1};0,\tau_{\mu 12}^{-1})$ means that *μ*_*i*1_ follows a zero mean normal distribution with variance $\tau_{\mu 12}^{-1}$. The first component corresponds to the genes that are not differentially expressed, e.g., *μ*_*i*1_ = *μ*_*i*2_ so for particular gene *i*, the two groups share the same variance. Likewise, the second component corresponds to those genes that are differentially expressed, e.g., *μ*_*i*1_ ≠ *μ*_*i*2_ so we assume independent normal priors with different variances for these two components. The Bayesian framework offers the flexibility required to specify the range of N/I distributions introduced in the previous section.

Therefore, in this content, the null and alternative hypothesis tests for the *i* ^*th*^ gene are defined as $H_{0}^{i}:\mu_{i1}=\mu_{i2}$ versus $H_{0}^{i}:\mu_{i1}\neq \mu_{i2}$.

We have used a Bayesian structure for the above mentioned model. To carry out Bayesian inference, the specification prior distributions for the unknown parameters is necessary. The prior distributions are given as *τ*_*ɛi*_ ∼ Γ(1, 0.005), *p* ∼ Dirichlet(*ϖ*); *ϖ* = (1, 1)′, *τ*_*μ*1_, *τ*_*μ*2_, *τ*_*μ*12_ ∼ Γ(1, 0.005), *i* = 1, 2, …, *n*. To obtain the t distribution, $U_{isr}∼\Gamma (\frac{v_{i}}{2},\frac{v_{i}}{2})$ and the prior distribution for *v*_*i*_ is *U*(0, 100). For the contaminated normal distribution, *λ*_*i*_, *γ*_*i*_ ∼ *U*(0, 1). For the slash distribution, *U*_*isr*_ ∼ *Beta*(*v*_*i*_,1) and the prior distribution for *v*_*i*_ is Γ(1, 0.005). Finally, for the Laplace distribution, $U_{isr}^{-1}∼exp(v_{i})$ and *v*_*i*_ ∼ Γ(1, 0.005). All the priors are chosen to be low-informative.

### 4.2 Multiple-group case

Sometimes there are more than two samples and identifying differences in the expression of the same gene between more than two samples may be of interest. Let there be *k* samples in the study; for example, in the BRCA data, there are three groups: BRCA1, BRCA2 and sporadic cases.

In some situations, tests for complicated null hypotheses can be developed from tests for simpler null hypotheses. The union-intersection method [22] of test construction might be useful when the null hypothesis is conveniently expressed as an intersection, say *H*_0_: *θ* ∈ ⋂_*γ* ∈ Γ_Θ_*γ*_, when Γ is an arbitrary index set that may be finite or infinite.

In the analysis of the gene expression data, the main null hypothesis for three groups is given by $H_{0}^{i}:\mu_{i1}=\mu_{i2}=\mu_{i3}.$ This null hypothesis can be considered as the following union-intersection test:

$$\begin{array}{c} H_{0}^{i}:\left(\mu_{i1}=\mu_{i2}\right)\cap \left(\mu_{i1}=\mu_{i3}\right)\cap \left(\mu_{i2}=\mu_{i3}\right). \end{array}$$ (2)

Therefore, having defined this hypothesis, one can implement all of the pairwise hypothesis tests. A gene is differentially expressed if at least one of the following hypothesis tests is rejected: ${H_{0}^{i}}^{\left(1\right)}:\mu_{i1}=\mu_{i2},$ ${H_{0}^{i}}^{\left(2\right)}:\mu_{i1}=\mu_{i3},$ ${H_{0}^{i}}^{\left(3\right)}:\mu_{i2}=\mu_{i3}.$

Now let there be *k* samples in the study. The main null hypothesis for *k* sample is given by $H_{0}^{i}:\mu_{i1}=\mu_{i2}=...=\mu_{ik}.$

This null hypothesis can be considered as the following union-intersection test: $H_{0}^{i}:(\mu_{i1}=\mu_{i2})\cap (\mu_{i1}=\mu_{i3})\cap ...\cap (\mu_{i,k-1}=\mu_{i,k}).$ Therefore, having defined this hypothesis, one can implement all of the $\frac{k(k-1)}{2}$ pairwise hypothesis tests. A gene is differentially expressed if at least one of the following hypothesis tests is rejected: ${H_{0}^{i}}^{\left(1\right)}:\mu_{i1}=\mu_{i2},$ ${H_{0}^{i}}^{\left(2\right)}:\mu_{i1}=\mu_{i3},$ …, ${H_{0}^{i}}^{\left(\frac{k(k-1)}{2}\right)}:\mu_{i,k-1}=\mu_{ik}.$ In order to address the problem of multiple comparisons when performing $\frac{k(k-1)}{2}$ pairwise tests of hypothesis, one could apply the Bonferroni correction. This means reducing the significance level at which each test is performed from the 5% level to 1% or even 0.1%.

Thus, in this context the structure of each hypothesis test is considered to be the same as in the two-group case. The prior distributions are the same as those which were considered in Section 4.1 and all the priors are chosen to be low-informative. The definition of Bayesian false discovery rate is given in S2 Appendix.

## 5 Applications

### 5.1 The Golub data

For detecting the differentially expressed genes, model (1) under the N/I distributional assumption is applied. In the Bayesian approach, two parallel MCMC chains with different initial values are run for 20,000 iterations each. Then, we have discarded the first 15,000 iterations as pre-convergence burn-in and retained 5,000 for the posterior inference. For checking convergence of the MCMC chains, the Gelman-Rubin diagnostic test [23] is used.

Table 1 shows the results for the Golub data. In this table, and other tables in this paper, N, T, SL, CN and Lap are used as abbreviations for the normal, the Student’s t, the slash, the contaminated normal and the Laplace distributions, respectively. A diagnostic tool to identify differentially expressed genes is to compute the posterior probabilities of *μ*_*i*1_ − *μ*_*i*2_ ≠ 0, *i* = 1, 2, …, *N*. Table 1 shows that the model which assumes a Laplace distribution detects more genes, 983, than models with other distributional assumptions at the *κ* = 0.5 posterior threshold [*P*(*μ*_1_ ≠ *μ*_2_∣Data) > *κ*]. At this threshold, bFDR, bFNR and bTNR of the model under the Laplace distributional assumption are smaller than those for the models assuming other distributions. At posterior thresholds 0.7, 0.9 and 0.95, although bFDR for the model under the Laplace distributional assumption is the smallest one, the best fitting model based on bTNR and bFNR is the model which assumes the t distribution. Therefore, a more conservative conclusion would be to choose the t distribution.

**Table 1:** Number of differentially expressed genes, bFDR, bTNR and bFNR in the Golub data. The values of bFDR, bTNR and bFNR for the best model are highlighted in **bold**.

*κ*	Model	No.	bFDR	bTNR	bFNR
0.50	N	880	0.1533	0.8328	0.1572
	CN	919	0.1502	0.8340	0.1597
	Lap	983	**0.1330**	**0.8510**	**0.1489**
	SL	820	0.1539	0.8498	0.1501
	T	814	0.1504	0.8503	0.1495
0.70	N	682	0.0801	0.7971	**0.2028**
	CN	695	0.0700	0.7935	0.2016
	Lap	783	**0.0632**	0.8107	0.1893
	SL	630	0.0775	0.8150	0.1849
	T	634	0.0777	**0.8189**	0.1810
0.90	N	459	0.0243	0.7452	0.2547
	CN	499	0.0216	0.7429	0.2418
	Lap	576	**0.0139**	0.7596	0.2403
	SL	432	0.0224	0.7684	0.2315
	T	429	0.0211	**0.7702**	**0.2297**
0.95	N	360	0.0110	0.7205	0.2794
	CN	405	0.0097	0.7203	0.2722
	Lap	503	**0.0057**	0.7398	0.2601
	SL	350	0.0104	0.7473	0.2526
	T	358	0.0113	**0.7518**	**0.2481**

An ROC curve can be plotted using bFPR versus bTPR for the possible posterior threshold *κ*. The values of bFPR and bTPR, using Eqs (1)–(3) in S1 Appendix, can be estimated using the following formulae:

$$\begin{array}{ccc} bFPR(r_{\Theta_{1}}^{\alpha }) & = & \frac{\sum_{i}^{}P\left(\theta_{i}\in \Theta_{0}|y_{i},r_{\Theta_{1}}^{\alpha }\left(y_{i}\right)\right)r_{\Theta_{1}}^{\alpha }\left(y_{i}\right)}{\sum_{i}^{}P\left(\theta_{i}\in \Theta_{0}|y_{i},r_{\Theta_{1}}^{\alpha }\left(y_{i}\right)\right)}, \end{array}$$ (3)

$$\begin{array}{ccc} bTPR(r_{\Theta_{1}}^{\alpha }) & = & \frac{\sum_{i}^{}P\left(\theta_{i}\in \Theta_{1}|y_{i},r_{\Theta_{1}}^{\alpha }\left(y_{i}\right)\right)r_{\Theta_{1}}^{\alpha }\left(y_{i}\right)}{\sum_{i}^{}P\left(\theta_{i}\in \Theta_{1}|y_{i},r_{\Theta_{1}}^{\alpha }\left(y_{i}\right)\right)}. \end{array}$$ (4)

This curve usually has a concave shape connecting the points (0, 0) and (1, 1).

**Fig 3:**
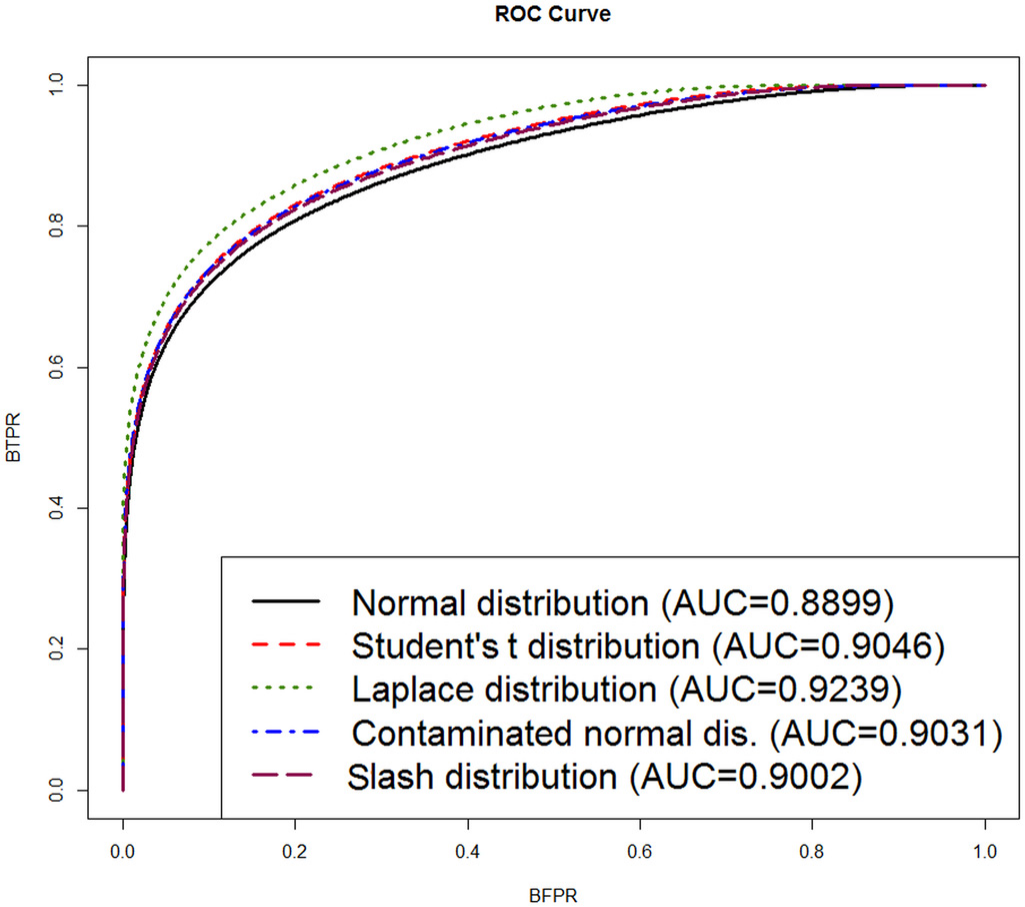
ROC curve and the area under the curve (AUC) under different distributional assumptions for the Golub data.

Fig 3 shows the ROC curves under different distributional assumptions. This figure shows that the ROC curve for the model under the Laplace distribution is higher than the ROC curve for the models under the other distributional assumptions. Also, in this figure the area under the curve (AUC) for each distribution is reported. This criterion shows that the model under the Laplace distributional assumption (with the highest AUC = 0.9239) is the best fitting model.

**Fig 4:**
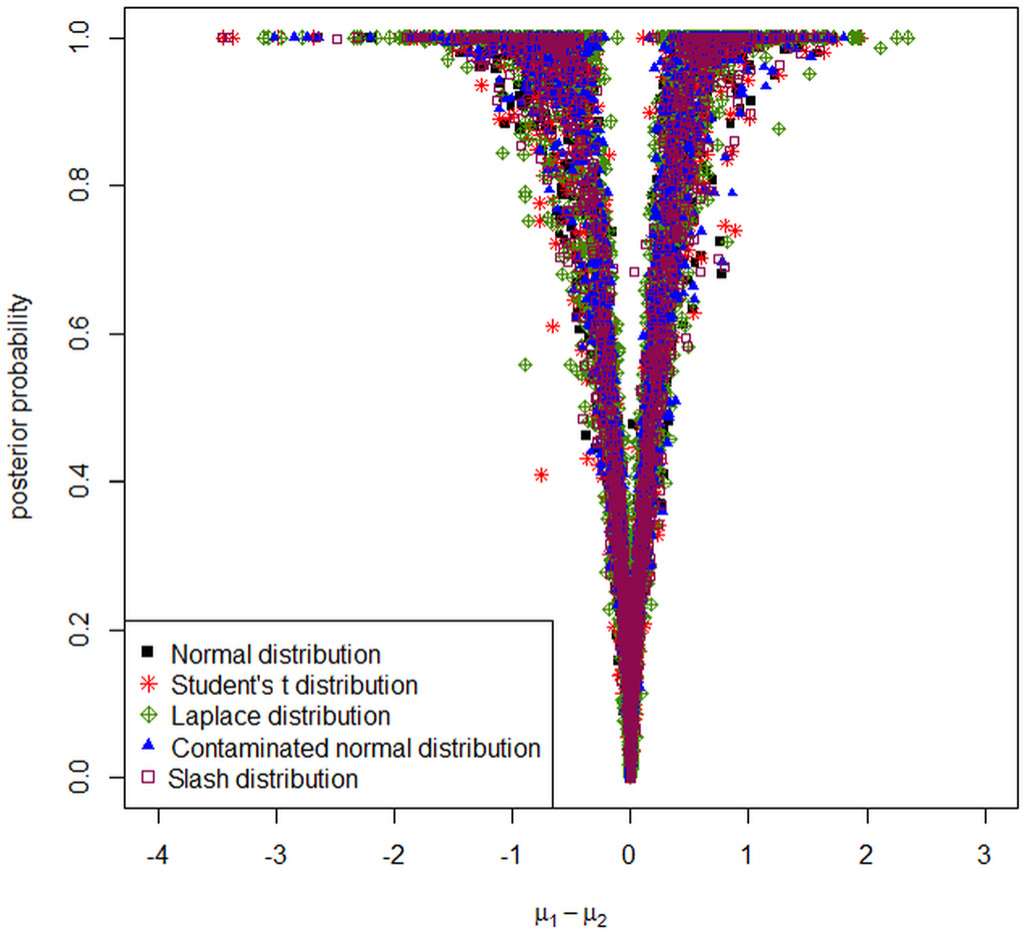
Posterior probabilities against the posterior differences between *μ*_1_ and *μ*_2_ from the model with different distributional assumptions for the Golub data.

Also, Fig 4 summarizes the posterior probabilities from the BRIN/IDGE method using different distributional assumptions for the errors. This figure plots the posterior probabilities of *μ*_1_ − *μ*_2_ ≠ 0 versus the posterior difference between the mean of the two groups. This figure shows that *μ*_1_−*μ*_2_s are shrinking towards zero and hence the genes with small values of *μ*_1_−*μ*_2_ have very low posterior probabilities of differential expression.

**Fig 5:**
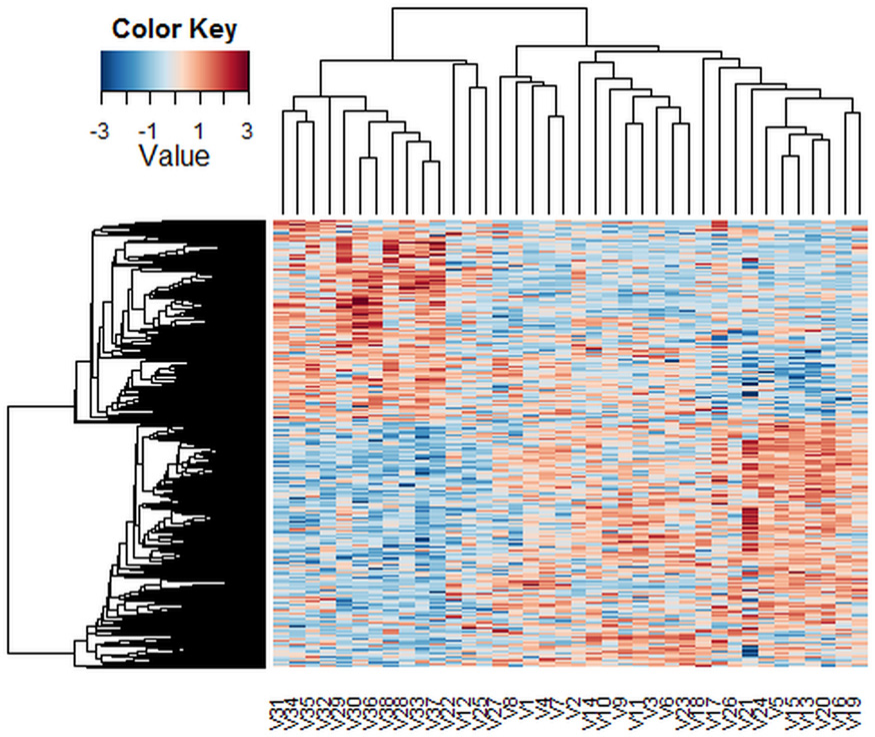
Heatmap of intensities of genes that were best differentiated between the two types of tumor for the Golub data.

Fig 5 shows the heatmap of the 983 genes that were best differentiated between the two types of tumor as determined by the Laplace distributional assumption for the errors at the 0.5 posterior threshold. This figure indicates that the detected genes may be divided in to two clusters, such that, some of the detected genes have higher levels of gene expression in the ALL sample and some of the detected genes have higher levels of gene expression in the AML sample. In comparison with existing methods, we use t-tests and Bonferroni-adjusted t-tests to detect the number of differentially expressed genes in the Golub data. The results show that, for the t-test, 1045 p-values are less than 0.05. The number of detected genes in the Bonferroni-adjusted t-tests is 98.

Significance Analysis of Microarrays (SAM) is a statistical method that has been developed by [7] for detecting differentially expressed genes. This method performs a two-class analysis using either a modified t-statistic or a (standardized) Wilcoxon rank statistic, and a multiclass analysis using a modified F-statistic. SAM uses regularized t-tests where the estimate of the standard deviation is regularized with a common estimate of the standard deviation and controls an estimate of the FDR value.

Let *X*_*ij*_, *j* = 1,…, *J* and *Y*_*ik*_, *k* = 1,…, *K*, *i* = 1, 2, …, *n* be the expression level of gene *i* under experimental conditions 1 and 2, respectively. In Table 2, the total number of genes declared significant is $\#\{i:|d_{\left(i\right)}-{\bar{d}}_{\left(i\right)}|>\Delta \}$, where $d_{\left(i\right)}=\frac{{\bar{X}}_{i}-{\bar{Y}}_{i}}{s(i)+s_{0}}$, ${\bar{X}}_{i}$ and ${\bar{Y}}_{i}$ are the averages of expression level for gene *i* under experimental conditions 1 and 2. Also, $s(i)=\sqrt{a\{\sum_{j=1}^{J}{\left(X_{ij}-{\bar{X}}_{i}\right)}^{2}+\sum_{k=1}^{K}{\left(Y_{ik}-{\bar{Y}}_{i}\right)}^{2}\}}$, *a* = (1/*J* + 1/*K*)/(*J* + *K* − 2). The constant *s*_0_ is chosen to minimize the coefficient of variation of *d*_(*i*)_, *i* = 1, 2, …, *n* (see [24], for more details). The results for this method are given in Table 2. In this table, “False” is the number of falsely called genes [7], “Called” is the number of genes called differentially expressed and FDR is the estimated FDR. In the SAM method, one has to choose the Δ value that is able to give the best compromise in terms of called genes, false genes and False Discovery Rate (FDR). In microarray analysis, it is very important to have statistically robust results, but we have to keep in mind that too small sized results are not able to describe the biological meaning of the experiment. In general, the choice of cut-off is subjective and there is no definition way of choosing it.

**Table 2:** The results of applying SAM to the Golub data. “False” is the number of falsely called genes, “Called” is the number of genes called differentially expressed and FDR is the estimated FDR.

	Δ	False	Called	FDR
1	0.1	2424.77	2739	0.44276
2	0.7	262.21	1248	0.10508
3	1.3	12.11	507	0.01195
4	1.8	0.74	210	0.00176
5	2.4	0.01	76	6.58e-05
6	3.0	0	15	0
7	3.6	0	5	0
8	4.1	0	2	0
9	4.7	0	2	0
10	5.3	0	0	0

The results show that, under 0.44276 for FDR, 2739 genes are detected as being differentially expressed (the results of using this method are obtained by using the SAM package in R). In our proposed method, the largest value for Bayesian FDR is 0.1330. As shown in Table 1, the number of differentially expressed genes in this case is 983. Thus, in Table 2, a more realistic FDR is 0.10508 with a Δ of 0.7 which results in 1248 genes being detected as differentially expressed genes. When the FDR is reduced further, Δ and the number of differentially expressed genes increased to 1248.

In Efron’s empirical model, a gene will be called differentially expressed if its posterior probability of being differentially expressed is larger than or equal to 1 − *α*. The results are shown in Table 3 and are obtained by using the SAM package in R. When 1 − *α* = 0.5, a FDR of 0.1593 results in 1466 genes being defined as differentially expressed. A more realistic FDR of 0.08855 (when 1−*α* = 0.7) results in 1131 genes being identified as differentially expressed. Also, the empirical Bayes lognormal-normal and gamma-gamma models, controlling the FDR at 10%, detect 650 and 861 genes, respectively. The results are obtained by using the EBarrays package in R.

**Table 3:** The results of applying Efron’s empirical model to the Golub data.

	1 − *α*	Number	FDR
1	0.5	1466	0.1593
2	0.7	1131	0.0885
3	0.9	698	0.0274
4	0.95	539	0.0142

### 5.2 The BRCA data

In this section, we analyzed the BRCA data using the model described in Section 4.2. For detecting differentially expressed genes, we have applied the union-intersection test using the BRIN/IDGE method.

**Fig 6:**
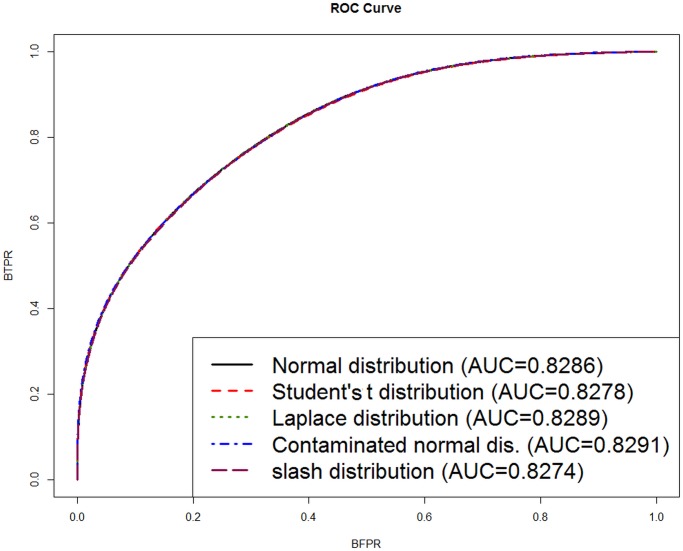
ROC curve and the area under the curve (AUC) under different distributional assumptions for the BRCA data.

The model comparison for this data set can be found in Table 4. This shows that different criteria, bFDR, bTNR and bFNR, for each *κ* value, have nearly the same number of differentially expressed genes. We conclude that, for the BRCA data, there is little to choose between the range of models making different distributional assumptions. Fig 6 shows the ROC curves and the AUCs for the models fitted under different distributional assumptions. This figure shows that all the models perform similarly well.

**Table 4:** Number of differentially expressed genes, bFDR, bTNR and bFNR in the BRCA data set. The values of bFDR, bTNR and bFNR for the best model are highlighted in **bold**.

*κ*	Model	No.	bFDR	bTNR	bFNR
0.50	N	685	0.2571	0.7865	0.2134
	CN	666	**0.2515**	**0.7868**	0.2331
	Lap	685	0.2563	0.7865	0.2134
	SL	694	0.2607	0.7859	0.2141
	T	692	0.2569	0.7867	**0.2132**
0.70	N	378	0.1341	0.7458	0.2541
	CN	369	**0.1244**	**0.7476**	**0.2524**
	Lap	380	0.1341	0.7460	0.2539
	SL	376	0.1352	0.7439	0.2561
	T	375	0.1288	0.7447	0.2552
0.90	N	163	0.0406	0.7078	0.2921
	CN	166	**0.0349**	**0.7111**	**0.2888**
	Lap	164	0.0406	0.7079	0.2921
	SL	158	0.0397	0.7055	0.2944
	T	162	0.0371	0.7067	0.2932
0.95	N	101	0.0187	0.6953	0.3047
	CN	110	**0.0147**	**0.6996**	**0.3003**
	Lap	101	0.0185	0.6951	0.3048
	SL	99	0.0195	0.6936	0.3063
	T	102	0.0159	0.6946	0.3054

Table 5 shows the estimates of the mixing probabilities for the five patterns of gene expression. This table indicates that all the models produce nearly the same probabilities *p*_*i*_ *s*, *i* = 1, 2, …, 5.

**Table 5:** Estimates of the mixing probabilities for the five patterns of gene expression for the BRCA data set. *p*_1_ : *μ*_1_ = *μ*_2_ = *μ*_3_, *p*_2_ : *μ*_1_ = *μ*_2_ ≠ *μ*_3_, *p*_3_ : *μ*_2_ ≠ *μ*_1_ = *μ*_3_, *p*_4_ : *μ*_1_ ≠ *μ*_2_ = *μ*_3_, *p*_5_ : *μ*_1_ ≠ *μ*_2_ ≠ *μ*_3_.

Model	*p*_1_	*p*_2_	*p*_3_	*p*_4_	*p*_5_
N	0.7876	0.0216	0.0006	0.1856	0.0040
CN	0.7935	0.0257	0.0000	0.1766	0.0040
Lap	0.7876	0.0220	0.0006	0.1857	0.0040
SL	0.7849	0.0236	0.0003	0.1872	0.0040
T	0.7877	0.0217	0.0006	0.1557	0.0043

**Fig 7:**
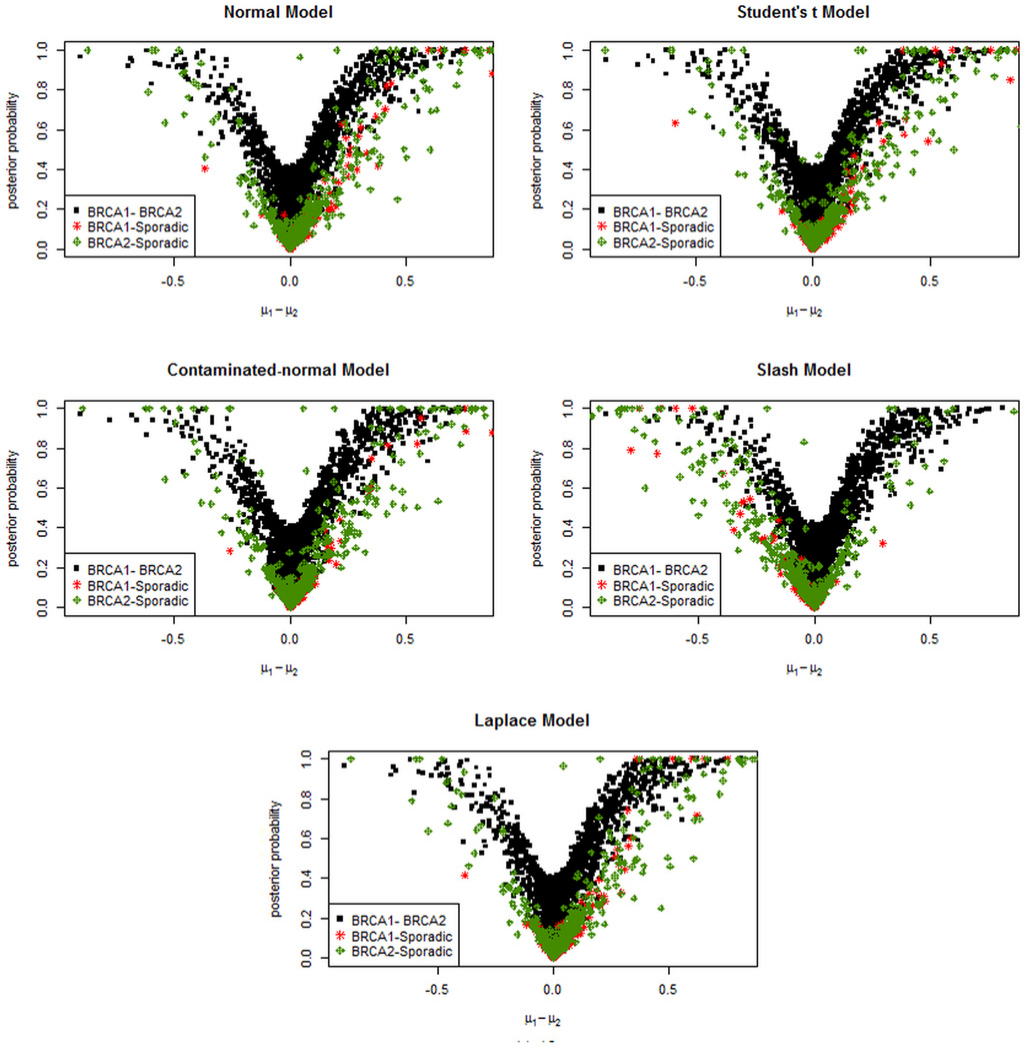
Posterior probabilities against the posterior differences between *μ*_1_ and *μ*_2_ from the model with different distributional assumptions for the BRCA data.

Fig 7 presents the posterior probabilities from the BRIN/IDGE method for each test using different distributional assumptions for the errors. This figure shows that differences in the mean have shrunk towards zero and hence have very low posterior probability of differential gene expression. This figure also shows that, the larger the difference in means *μ*_1_−*μ*_2_ the larger are the posterior probabilities.

## 6 Simulation Studies

In this section, some simulation studies are conducted in order to illustrate the performance of our proposed methodology. In each simulation study, *N* simulated genes are generated and *M* iterations are performed.

To record a case identified by posterior probabilities as being differentially expressed, we define the following indicator variables: $I_{ik}^{Method}=\{\begin{array}{cc} 0 & \mu_{i1}=\mu_{i2}\\ 1 & o.w. \end{array}$ such that $I_{ik}^{Method}=1$ if *P*(*μ*_*i*1_ ≠ *μ*_*i*2_∣***y***) > 0.5. For *i* = 1, 2, …, *N* and *k* = 1, 2, …, *M*, also, for the real situation

$$\begin{array}{c} I_{ik}^{Real}=\{\begin{array}{cc} 0 & \mu_{i1}=\mu_{i2}\\ 1 & o.w. \end{array} \end{array}$$ (5)

In the generated data set, we let 100*p*% of data have different means and 100(1 − *p*)% of the generated data have the same means (see subsections 6.1 and 6.2 for more details). So, let *p* be the proportion of differentially expressed genes. The true positive rate (TPR), the false positive rate (FPR) and true discovery rate (TDR) for the *k* ^*th*^ iteration can be calculated as follows:

$$\begin{array}{ccc} TPR_{k} & = & \frac{\sum_{i=1}^{N}I_{ik}^{Real}I_{ik}^{Method}}{N\times p}, \end{array}$$ (6)

$$\begin{array}{ccc} FPR_{k} & = & \frac{\sum_{i=1}^{N}\left(1-I_{ik}^{Real}\right)I_{ik}^{Method}}{N\times (1-p)}, \end{array}$$ (7)

$$\begin{array}{ccc} TDR_{k} & = & \frac{\sum_{i=1}^{N}I_{ik}^{Real}I_{ik}^{Method}}{\sum_{i=1}^{N}I_{ik}^{Method}}. \end{array}$$ (8)

Averaging across all iterations, we have:

$$\begin{array}{ccc} T\bar{P}R & = & \frac{1}{M}\sum_{k=1}^{M}TPR_{k}, \end{array}$$ (9)

$$\begin{array}{ccc} F\bar{P}R & = & \frac{1}{M}\sum_{k=1}^{M}FPR_{k}, \end{array}$$ (10)

$$\begin{array}{ccc} T\bar{D}R & = & \frac{1}{M}\sum_{k=1}^{M}TDR_{k}. \end{array}$$ (11)

A receiver operating characteristic (ROC) curve is a plot of FPR versus TPR for the possible cutoffs *κ* [*P*(*μ*_*i*1_ ≠ *μ*_*i*2_∣***y***) > *κ*]. An ROC curve is a two-dimensional depiction of classifier performance [25]. A common method of comparing the classifiers is the area under the ROC curve (often referred to as the AUC). In our simulation study, we calculate *AUC*_*k*_, *k* = 1, 2, …, *M* for each iteration and we report $A\bar{U}C=\frac{1}{M}\sum_{k=1}^{M}AUC_{k}$. *AUC*_*k*_ and (consequently $A\bar{U}C$) is a portion of the area of the unit square; its value will always lie between 0 and 1. The larger the value of $A\bar{U}C$, the better is the performance of the classifier.

In order to perform a Bayesian analysis, we need a number of iterations for each gene including a number for pre-convergence burn-in. In this simulation study, the MCMC chains are run for 15,000 iterations each. Then, we discarded the first 10,000 iterations as pre-convergence burn-in and retained 5,000 for the posterior inference. More details of the approaches can be found in the following sub-sections.

### 6.1 Simulation study 1

In this section, a simulation study is conducted to check the performance of the proposed BRIN/IDGE method, when the real distribution of the gene expression is the contaminated normal distribution. This distribution has a bimodal form which is commonly found in gene expression data. For this purpose, a sample with *N* = 1000 genes is evaluated and *M* = 100 iterations are performed. We consider the model *Y*_*isr*_ = *μ*_*is*_ + *ɛ*_*isr*_ such that *ɛ*_*isr*_ ∼ *CN*(0, *σ*_*i*_, ***ν***_*i*_), *r* = 1, 2, …, *n*_*s*_ and *s* = 1, 2. To generate the simulated data sets, we fix *μ*_*is*_ = 14, *σ*_*i*_ = 1 and ***ν***_*i*_ = (*λ*_*i*_, *γ*_*i*_)′, *λ*_*i*_ = 0.1 and the two values for *γ*_*i*_: 0.10 and 0.25. Also, *n*_1_ = 27 and *n*_2_ = 11 are considered.

To verify how the method behaves when the control group moves away from the treatment group, we choose randomly 5% of the genes in the first group. These observations are generated by the location parameter *μ*_*i*1_ + *δ* where *δ* ∈ {3, 5}.

The results of this simulation study are reported in Table 6. This table presents the results of $T\bar{P}R$, $F\bar{P}R$, $T\bar{D}R$ and $A\bar{U}C$ under different distributional assumptions. These results show the good performance of the robust models, in particular the model which assumes the contaminated normal distribution for the errors. This table shows that the normal distribution is not able to detect the differentiated genes. Also, as *δ* is increased from 3 to 5, the ability of all distributions to detect differentiated genes is improved, although the reliability of the robust distributions is greater than that of the normal one. The results show that, as *γ* is increased from *γ* = 0.1 to *γ* = 0.25, most of the comparison criteria ($T\bar{P}R$, $T\bar{D}R$ and $A\bar{U}C$) for the normal distribution are severely reduced in value, but the robust distributions have better ability to detect differentially expressed genes.

**Table 6:** Results of simulation study for *n*_1_ = 27 and *n*_2_ = 11. Data are generated by the contaminated normal distributional assumption for the errors. The values of $A\bar{U}C$ for the best model are highlighted in **bold**.

		*δ* = 3				*δ* = 5			
*γ*		$T\bar{P}R$	$F\bar{P}R$	$T\bar{D}R$	$A\bar{U}C$	$T\bar{P}R$	$F\bar{P}R$	$T\bar{D}R$	$A\bar{U}C$
0.10	N	0.2750	0.0000	1.0000	0.7013	0.7299	0.0005	0.9928	0.9276
	CN	0.9952	0.0033	0.9727	**0.9988**	1.0000	0.0016	0.9863	**0.9994**
	Lap	0.8551	0.0033	0.9708	0.9027	0.9951	0.0016	0.9863	0.9994
	SL	0.9599	0.0022	0.9813	0.9773	0.9851	0.0027	0.9772	0.9979
	T	0.9801	0.0044	0.9643	0.9826	1.0000	0.0038	0.9689	0.9983
0.25	N	0.0714	0.0000	0.3181	0.3751	0.3052	0.0005	0.7819	0.7915
	CN	0.8952	0.0010	0.9909	**0.9659**	0.9947	0.0029	0.9768	**0.9982**
	Lap	0.3571	0.0005	0.9920	0.6351	0.8947	0.0011	0.9893	0.9564
	SL	0.8238	0.0021	0.9829	0.9108	0.9157	0.0029	0.9750	0.9864
	T	0.8476	0.0010	0.9909	0.9364	0.9842	0.0005	0.9952	0.9947

### 6.2 Simulation study 2

In this subsection, as in subsection 7.1, a simulation study is conducted to check the performance of the BRIN/IDGE, method as well as the usual normal model, when data are generated from the symmetric t distribution. For this purpose, the model defined in Section 4.1, with *ɛ*_*isr*_ ∼ *t*(0, *σ*_*i*_, *ν*_*i*_), *σ*_*i*_ = 1 and *ν*_*i*_ = 2, is used. As in Section 7.1, *μ*_*is*_ = 14 (*δ* ∈ {3, 5}) is considered.

The results of this simulation study are summarized in Table 7. These results show that the performance of the robust models for detecting differentially expressed genes is better than that of the normal one. Also, as *δ* is increased from 3 to 5, the ability of the distributions to detect differentially expressed genes has improved. The results show that, when *δ* = 3, the slash distributional assumption for the error provides the best performance among the models but, for *δ* = 5, the Student’s t distribution, performs the best.

**Table 7:** Results of simulation study for *n*_1_ = 27 and *n*_2_ = 11. Data are generated by t distributional assumption for the errors. The values of $A\bar{U}C$ for the best model are highlighted in **bold**.

	*δ* = 3				*δ* = 5			
	$T\bar{P}R$	$F\bar{P}R$	$T\bar{D}R$	$A\bar{U}C$	$T\bar{P}R$	$F\bar{P}R$	$T\bar{D}R$	$A\bar{U}C$
N	0.6667	0.0158	0.7470	0.8348	0.9556	0.0181	0.7802	0.9548
CN	0.7555	0.0152	0.7515	0.8412	0.9777	0.0198	0.7367	0.9653
Lap	0.8444	0.0081	0.8522	0.8832	0.9556	0.0152	0.7879	0.9674
SL	0.7111	0.0105	0.8018	**0.9204**	0.9777	0.0175	0.7705	0.9679
T	0.7556	0.0175	0.7010	0.8732	1.0000	0.0140	0.8022	**0.9892**

## 7 Conclusion

In this paper, we have proposed the use of robust models for detecting differentially expressed genes. For this purpose, some powerful distributions that are known as normal/independent (N/I) distributions are used. These distributions include the Student’s t, the slash, the contaminated normal and the Laplace distributions. We have applied our proposed approach in two-group and multiple-group scenarios. A union-intersection test is used for detecting differential gene expression in the multiple-group case. The source code written in R (R2OpenBUGS package) is available on “*bs.ipm.ac.ir/softwares/BRIN/index.jsp*”.

To investigate the performance of our proposed approach, some simulation studies have been performed. Also, two real data sets have been analyzed where the models have been compared using bFDR, bTNR, bFNR and area under the ROC curve. We have demonstrated the flexibility of robust models in identifying differentially expressed genes. In other words, a well performing model in the class of N/I models should be identified in the light of the data. As an extension, one may consider the use of the skew-normal/independent family of mdels [26] to analyze gene expression data.

## Supporting Information

S1 Appendix (PDF)

S2 Appendix (PDF)
