# Supplementary material for: Robust Modeling of Differential Gene Expression Data Using Normal/Independent Distributions: A Bayesian Approach
Source: PLoS One. 2015 Apr 24;10(4):e0123791. doi: 10.1371/journal.pone.0123791 (PMC4409222; doi:10.1371/journal.pone.0123791)
Supplement: S1 Appendix — (PDF) [file pone.0123791.s001.pdf]

Supporting Information

Robust Modeling of Differential Gene Expression Data Using Normal/Independent Distributions: A Bayesian Approach

Mojtaba Ganjali[1,2*], Taban Baghfalaki[1,3] and Damon Berridge[4]

1 School of Biological Science, Institute for Research in Fundamental Sciences (IPM), Tehran, Iran.

2 Department of Statistics, Faculty of Mathematical Sciences, Shahid Beheshti University, Tehran, Iran.

3 Department of Statistics, Faculty of Mathematical Sciences, Tarbiat Modares University, Tehran, Iran.

4 Farr Institute-CIPHER, College of Medicine, Swansea University, Swansea, Wales, U.K.

∗ Corresponding author
E-mail: m-ganjali@sbu.ac.ir (MG)

Appendix S1: Members of family of N/I distributions

Student's t distribution

The univariate Student's t distribution with location parameter μ, scale parameter σ and $\nu > 0$ degrees of freedom [1-2], $Y \sim t(\mu, \sigma, \nu)$, is an alternative to the normal distribution and has been frequently used in the literature; for example, see [3-6]. In the N/I distributional framework, if $U \sim \Gamma(\nu/2, \nu/2)$ which is a gamma distribution with mean 1, then $Y \sim t(\mu, \sigma, \nu)$.

Slash distribution

The slash distribution [7] is another N/I distribution. Its pdf is given by $f(y|\mu, \sigma, \nu) = \nu \int_0^1 u^{\nu-1}\phi(y|\mu, u^{-1}\sigma)\, du$, which has heavier tails than those of the normal distribution. It includes the normal distributions as a special case when $\nu \to \infty$. For a slash distribution, we shall use the notation $Y \sim SL(\mu, \sigma, \nu)$. In the N/I distributional framwork, $G(u; \nu)$ has density $g(u; \nu) = \nu u^{\nu-1} I(0, 1)$, i.e. $g(.; \nu)$ is a beta distribution with parameter $(\nu, 1)$.

Contaminated normal distribution

The contaminated normal distribution [8] is more applicable for modeling symmetric data with outlying observations. Its pdf is given by $f(y|\mu,\sigma,\boldsymbol{\nu}) = \gamma\phi(y|\mu,\frac{\sigma}{\lambda}) + (1-\gamma)\phi(y|\mu,\sigma)$. The parameter γ represents the percentage of outliers, while λ may be interpreted as a scale factor. The notation $Y \sim CN(\mu,\sigma,\boldsymbol{\nu})$ will be used for the contaminated normal distribution where $\boldsymbol{\nu} = (\lambda,\gamma)'$. If in the N/I distributional framework, the probability density of U, given the two-component parameter vector $\boldsymbol{\nu}$, is $g(u;\boldsymbol{\nu}) = \begin{cases} \gamma, & u = \lambda \\ 1-\gamma, & u = 1 \end{cases}$ where, $0 < \lambda < 1$ and $0 \leq \gamma < 1$, then we have $Y \sim CN(\mu,\sigma,\boldsymbol{\nu})$. Some applications of the contaminated normal distribution are given in [3].

Laplace or double exponential distribution

The Laplace or double exponential distribution [9-10] is another member of the N/I family of distributions. To define this robust distribution in he N/I distributional framework, U has an inverse gamma distribution, i.e., the density function of U is given by $g(u;\nu) = \frac{1}{u^2\nu}\exp\left(-\frac{1}{u^2\nu}\right)$, and $\sigma = 1$. Therefore, when e is a standard normal variable, then Y, generated as $Y = \mu + e/\sqrt{u}$, will have pdf $f(y|\mu,\nu) = \frac{1}{2}\sqrt{\frac{2}{\nu}}\exp\left(-\sqrt{\frac{2}{\nu}}|y-\mu|\right)$. This equation is recognized as the pdf of the Laplace distribution centered at μ. We denote this distribution by $Y \sim Lap(\mu,\nu)$.

References

1. Kotz S, Balakrishnan N, Johnson NL. Continuous multivariate distributions : Models and Applications. John Wiley & Sons; 2000.

2. Kotz S, Nadarajah S. Multivariate T-Distributions and Their Applications. Cambridge University Press; 2004.

3. Little RJA. Robust Estimation of the mean and covariance matrix from data with missing values . Applied Statistics 1988; 37: 23-38.

4. Lange KL, Little JA, Taylor MGJ. Robust statistical modeling using the t distribution. Journal of the American Statistical Association 1989; 84: 881-896.

5. Geweke J. Bayesian treatment of the independent Student-t linear model. Journal of Applied Economic 1993; 8: 19-40.

6. Pinheiro JC, Liu CH, Wu YN. Efficient algorithms for robust estimation in linear mixed-effects models using the multivariate t distribution. Journal of Computational and Graphical Statistics 2001; 10: 249-276.

7. Wang J, Genton M. The multivariate skew-slash distribution. Journal of Statistical Planning and Inference 2006; 136: 209–220.

8. Tukey JW. A survey of sampling from contaminated distribution. Paper 39 (p.448-485) in Contributions to probability and statistics (edited by I. Olkin, et. al.) Stanford University Press; 1960.

9. Andrews DF, Mallows CL. Scale mixtures of normal distributions. Journal of the Royal Statistical Society-Series B 1974; 36: 99–102.

10. Eltoft T, Kim T, Lee T. On the multivariate Laplace distribution. IEEE Signal Processing Letters 2006; 13(5): 300–303.
