# Supplementary material for: Robust Modeling of Differential Gene Expression Data Using Normal/Independent Distributions: A Bayesian Approach
Source: PLoS One. 2015 Apr 24;10(4):e0123791. doi: 10.1371/journal.pone.0123791 (PMC4409222; doi:10.1371/journal.pone.0123791)
Supplement: S2 Appendix — (PDF) [file pone.0123791.s002.pdf]

Supporting Information

Robust Modeling of Differential Gene Expression Data Using Normal/Independent Distributions: A Bayesian Approach

Mojtaba Ganjali[1,2*], Taban Baghfalaki[1,3] and Damon Berridge[4]

1 School of Biological Science, Institute for Research in Fundamental Sciences (IPM), Tehran, Iran.

2 Department of Statistics, Faculty of Mathematical Sciences, Shahid Beheshti University, Tehran, Iran.

3 Department of Statistics, Faculty of Mathematical Sciences, Tarbiat Modares University, Tehran, Iran.

4 Farr Institute-CIPHER, College of Medicine, Swansea University, Swansea, Wales, U.K.

∗ Corresponding author
E-mail: m-ganjali@sbu.ac.ir (MG)

Appendix S2: Bayesian False Discovery Rate

Suppose that $\theta_i, i = 1, 2, ..., n$ is the parameter of interest for gene i and let $\theta_i \in \Theta = (\Theta_0 \cup \Theta_1)$. The null hypothesis for gene i is defined as $H_0^{(i)} : \theta_i \in \Theta_0$ and the alternative is $H_1^{(i)} : \theta_i \in \Theta_1$. The Bayesian decision rule rejects $H_0^{(i)}$ if $P(\theta_i \in \Theta_1|y_i) > 1 - \alpha$, for some user-prespecified α and where Y_i are the observed data. In a Bayesian paradigm, the false discovery rate (FDR) is a measure of the posterior belief of the event $\theta_i \in \Theta_0$ given that $H_1^{(i)}$ was chosen [1]. Let $r^{\alpha}_{\Theta_1}(\boldsymbol{y}_i) = 1$ if $H_1^{(i)}$ is chosen and $r^{\alpha}_{\Theta_1}(\boldsymbol{y}_i) = 0$ otherwise. The Bayesian FDR (bFDR, [2-3]) is defined as:

$$bFDR(r^{\alpha}_{\Theta_1}) = \frac{\sum_i P(\theta_i \in \Theta_0|y_i, r^{\alpha}_{\Theta_1}(y_i))r^{\alpha}_{\Theta_1}(y_i)}{\sum_i r^{\alpha}_{\Theta_1}(y_i)}. \quad (1)$$

In order to compute this criterion, we refer to the first equation of the main file. Two scenarios exist based on this equation:

1. Two groups are not differentially expressed, that is $\mu_{i1} = \mu_{i2}$.

2. Two groups are differentially expressed, that is $\mu_{i1} \neq \mu_{i2}$.

The Bayesian false discovery rate can be computed by considering the genes for which $r^{\alpha}_{\Theta_1}(y_i) = 1$, that is the genes under H^g_1. In the denominator of the bFDR, the number of genes for which rejected H^g_0 at α level is computed and in the numerator of the bFDR, the sum of the posterior probabilities of $\theta_i \in \Theta_0$ for these genes is considered. Also, the Bayesian true negative rate (bTNR) is defined as:

$$bTNR(r^{\alpha}_{\Theta_1}) = \frac{\sum_i P(\theta_i \in \Theta_0 | y_i, r^{\alpha}_{\Theta_1}(y_i))(1 - r^{\alpha}_{\Theta_1}(y_i))}{\sum_i (1 - r^{\alpha}_{\Theta_1}(y_i))}. \quad (2)$$

As in the competition of bFDR, the denominator of the bTNR is the number of genes who rejected H^g_1 at α level and the numerator of the bTNR is the sum of posterior probabilities of $\theta_i \in \Theta_0$ for these genes.

In the same manner, the Bayesian false negative rate (bFNR) can be defined as:

$$bFNR(r^{\alpha}_{\Theta_1}) = \frac{\sum_i P(\theta_i \in \Theta_1 | y_i, r^{\alpha}_{\Theta_1}(y_i))(1 - r^{\alpha}_{\Theta_1}(y_i))}{\sum_i (1 - r^{\alpha}_{\Theta_1}(y_i))}. \quad (3)$$

The computation of bFNR is the same as that of bFDR and bTNR.

Notice that these rates depend on the rejection decision rule and consequently upon the choice of (Θ_1, α). In practice, the set Θ_1 is fixed and the decision rule is allowed to vary with the choice of α. In the following, for differential gene expression with two groups, the null hypothesis for gene i is defined as $H^{(i)}_0 : \mu_{i1} = \mu_{i2}$ and the alternative is $H^{(i)}_1 : \mu_{i1} \neq \mu_{i2}$. In the multiple groups scenario, the null hypothesis for gene i is defined as $H^{(i)}_0 : \mu_{i1} = \mu_{i2} = ... = \mu_{ik}$ and the alternative is $H^{(i)}_1 : \mu_{ik} \neq \mu_{ik'}$ for some $k \neq k'$.

References

1. Mallick BK, Gold DL, Baladandayuthapani V. Bayesian analysis of gene expression data. Wiley, Chichester, U.K. 2009.

2. Muller P, Parmigiani G, Robert C, Rousseau J. Optimal sample size for multiple testing: the case of gene expression microarray. *Journal of the American Statistical Association* 2004; 99: 990-1001.

3. Whittemore AS. A Bayesian false discovery rate for multiple testing. Journal of Applied Statistics 2007; 34: 1-9.
